# Validation of Responsiveness of Physicians Scale (ROP-Scale) for hospitalised COVID-19 patients in Bangladesh

**DOI:** 10.1186/s12913-022-08413-4

**Published:** 2022-08-15

**Authors:** Taufique Joarder, Mohammad Aminul Islam, Md Shariful Islam, Shabnam Mostari, Md. Tanvir Hasan

**Affiliations:** 1 Public Health Foundation, Bangladesh, Dhaka, Bangladesh; 2 Department of Media Studies and Journalism, University of Liberal Arts Bangladesh, Dhaka, Bangladesh; 3 Aspire to Innovate (a2i) Programme, ICT Division, Government of Bangladesh, Dhaka, Bangladesh; 4 BRAC James P Grant School of Public Health, BRAC University, Dhaka, Bangladesh

**Keywords:** Responsiveness of physicians, Patient satisfaction, Psychometric evaluation, Scale validation, Bangladesh

## Abstract

**Background:**

Responsiveness of Physicians (ROP) is defined as the social actions by physicians aimed at meeting the legitimate expectations of healthcare users. Even though patients’ expectations regarding ROP have increased during the COVID-19 pandemic, the psychometrically-validated ROP-Scale is difficult to apply in hospital settings. The goal of this study is to validate the existing ROP-Scale to measure the responsiveness of hospital physicians during the ongoing COVID-19 pandemic in Bangladesh.

**Methods:**

We conducted a cross-sectional phone survey involving 213 COVID-19 hospital patients, randomly selected from the government database. We applied the Delphi method for content validity, exploratory and confirmatory factor analyses for construct validity, Cronbach’s alpha and corrected item-total correlation for internal consistency reliability, and Pearson’s correlation between the scale and overall patient satisfaction for concurrent validity.

**Results:**

After removing survey items based on data sufficiency, collinearity, factor loading derived through exploratory factor analysis, and internal consistency, the final version of the COVID-19 ROP-Scale consisted of 7 items, grouped under Informativeness, Trustworthiness and Courteousness domains. The confirmatory factor analysis supported the three domains with acceptable model fit [Root mean squared error of approximation (RMSEA) = 0.028, Comparative fit index (CFI) = 0.997, Tucker-Lewis index (TLI) = 0.994)]. The corrected item-total correlation ranged between 0.45 and 0.71. Concurrent validity was ascertained by the high correlation (0.84) between patient satisfaction and the COVID-19 ROP-Scale. Based on the mean domain score, the highest- and the lowest-scoring responsiveness domains were ‘Trustworthiness’ (7.85) and ‘Informativeness’ (7.28), respectively, whereas the highest- and the lowest-scoring items were ‘Not being involved in illegal activities’ (7.97), and ‘Service-oriented, not business-like attitude’ (6.63), respectively.

**Conclusions:**

The 7-item COVID-19 ROP-Scale was demonstrated to be feasible, valid, and internally consistent. Therefore, its application can help amend past mistakes in health service provision and improve care for the hospitalised COVID-19 patients or other patients suffering from similar conditions. This study can contribute to the national decision-making regarding hospital care, open up further avenues in the health policy and system research, and eventually improve the quality of care provided to Bangladeshi patients seeking hospital services. Moreover, findings yielded by this study can be incorporated into doctors’ medical education and in-service training.

**Supplementary Information:**

The online version contains supplementary material available at 10.1186/s12913-022-08413-4.

## Introduction

The aim of health systems is delivering patient care in a timely manner. To this end, Responsiveness of Physicians (ROP) refers to the social actions by physicians to meet the legitimate expectations of healthcare users [1]. This is particularly important in a pandemic situation when patient load is high, and physicians are strained both physically and mentally [2]. Failure to respond to patent needs is associated with hesitation to follow-up [3] and even treatment discontinuation [4].

The ongoing COVID-19 pandemic has brought multiple challenges for both healthcare providers and patients, as it has increased anxiety, distrust, and uncertainty [5, 6], necessitating greater sensitivity to patient needs. As a result, ROP has gained attention of both policymakers and scholars from various disciplines such as public health, social psychology, social medicine, healthcare communication, and media studies.

Yet, measuring ROP in times of crises can be challenging, necessitating a reliable and objective scale pertaining to different aspects of physicians’ responsiveness and help them mend the gaps and improve their performance. During the pandemic, various disturbing allegations have been made against the physicians by COVID-19 patients in many low- and middle-income countries, including Bangladesh, ranging from medical negligence to outright denial to provide medical services. By providing physicians with an evidence-based set of activities or behaviours during the consultation process, they would be better prepared to care for patients suffering from COVID-19, thus improving public trust in healthcare services [6].

Although a psychometrically-validated ROP-Scale with five sub-scales and 34 items characterised by high internal consistency (alpha coefficient of 0.91) and inter-rater reliability (Intraclass Correlation Coefficient of 0.64 for individual rater’s reliability and 0.84 for average reliability scores) exists [7], it not only requires structured observation but was developed in the rural outpatient context of Bangladesh. Thus, it was not intended for hospital settings or times of infectious disease outbreaks. As most COVID-19 patients receive care from the inpatient department of a hospital or clinic and require appropriate care and observation, the ROP-Scale needs to be validated for this setting. Thus, as a part of the present study, the existing ROP-Scale was validated using psychometric techniques such as exploratory and confirmatory factor analyses to measure the responsiveness of inpatient hospital physicians during the COVID-19 pandemic in Bangladesh.

## Methods

### Design

The data gathered via a cross-sectional phone survey involving 213 persons who received COVID-19 care in July 2020 from any COVID-19-designated hospitals designated by the Government of Bangladesh (GoB) were subjected to psychometric analyses to validate the existing ROP-Scale [7].

### Participants

The study participants were recruited from both public and private COVID-19-designated hospitals across Bangladesh and were identified through the database of COVID-19 patients provided by the Aspire to Innovate (A2i) Programme, Information and Communication Technology (ICT) Division of the GoB that contains patient’s name, disease outcome, date of admission and discharge, hospital name, and contact information. We sampled potential participants from the database and extracted the contact information for the survey which was conducted over the phone due to the pandemic.

Only adult patients (aged ≥18 years) who were treated in one of the COVID-19-designated hospitals in July 2020 whose correct phone number was available in the database were eligible for participation. Those that did not consent to participate or could not be reached after calling three times at different time points were excluded from the sample.

### Content validity

Item clarity and content validity were established using a modified Delphi technique. The first author, who is a health policy and systems researcher with expertise in psychometrics, served as the facilitator. He convened the panel of experts (all of whom are the article co-authors) comprising of two public health physicians, a communication expert, and a biostatistician experienced in psychometrics, and provided them with the existing ROP-Scale, along with the guidelines for refining its 34 items through three iterations. The aim was to ensure that the scale items are [1] comprehendible over the phone, [2] applicable to the COVID-19 inpatient hospital context, and [3] appropriate for a questionnaire survey (the original ROP-Scale items were designed for structured observation and the four response categories were anchored in an outpatient consultation scenario), and that [4] the whole questionnaire is short enough not to discourage voluntary participation.

### Sample size and sampling techniques

The sample size was determined based on the 10:1 “n to p ratio” [8, 9], where ‘n’ is the minimum sample size, and ‘p’ is the number of items. Since the initial tool consisted of 20 items, the required sample size was 200. We used the RAND function in MS Excel (MS Office Professional Plus 2016) to randomly select the 250 patients based on simple random sampling technique. Anticipating a 20% non-response rate, as the survey was to be conducted over the phone and the patients may be weak and reluctant to participate after recovering from COVID-19, we approached 250 patients and obtained 213 valid responses.

### Data collection instruments and procedures

The survey questionnaire consisted of two parts: (1) Socio-demographic characteristics (age, gender, education, occupation, current residence, religion, marital status, and monthly income), and (2) ROP-Scale items. From the database, we also extracted the location and type of hospitals (public or private) in which the patients received care, duration of hospital stay, and disease severity. The ROP-Scale section was further subdivided into seven sub-sections, namely (1) Beginning part, (2) History taking, (3) Examination, (4) Prescribing, (5) Explaining, (6) Leaving, and (7) Throughout consultation. Participants were also asked to rate their level of overall satisfaction with the services received from the doctors in the hospital. The 20-item draft tool was pilot-tested on five non-sampled patients and the language was improved for intelligibility based on their feedback. The original ROP-Scale items and the draft COVID-19 ROP-Scale items used for data collection in this research are shown in Table 1.

**Table 1:** ROP-Scale’s original items and draft items for this study

Name of Sub-scales or Domains	Definition	Items in Original ROP-Scale	Items Retained in the Draft COVID-19 ROP-Scale
Friendliness	How a physician communicates with a patient	1. Asking patient’s name 2. Engaging in social talks 3. Asking about patient’s family 4. Friendliness 5. Giving courage and reassurance 6. Sense of humour	1. Engaging in social talks 2. Friendliness 3. Giving courage and reassurance 4. Sense of humour
Respecting	How a physician explicitly shows respect to a patient	1. Greetings by physician 2. Showing respect explicitly 3. Listening to patient’s complaints completely 4 Listening to patient’s complaints attentively 5. Examining the patient with care 6. Encouraging patient to ask questions 7. Listening attentively to patient’s questions 8. Closing salutation by physician 9. Non-verbal communication by physician 10. Compassionately touching the patient by physician	1. Greetings by physician 2. Showing respect explicitly 3. Listening to patient’s complaints attentively 4. Examining the patient with care 5. Encouraging patient to ask questions
Informing and guiding	How a physician empowers a patient	1. Suggestions on disease prevention and health promotion in general 2. Facilitating follow-up 3. Quantity of issues explained and the quality of explanation 4. Quantity of issues explained 5. Asking patient if s/he understood the explanation 6. Explaining the cause of disease to the patient 7. Explaining the diagnosis of disease to the patient 8. Explaining the prognosis of disease to the patient 9. Explaining the treatment to the patient 10. Explaining the preventive aspects to the patient	1. Facilitating follow-up 2. Explaining the cause of disease to the patient 3. Explaining the diagnosis of disease to the patient 4. Explaining the prognosis of disease to the patient 5. Explaining the treatment to the patient 6. Explaining the preventive aspects to the patient
Gaining trust	How a physician may gain trust of the patients, or refrains from doing something that may breach trust of the patients	1. Earning trust of patients 2. Service oriented, not business-like attitude 3. Not using jargon 4. Not being involved in illegal activities	1. Service oriented, not business-like attitude 2. Not being involved in illegal activities
Financial sensitivity	Understanding financial need of the patients and providing support if needed, going beyond the consultation	1. Considering the socio-economic status of the patient 2. Trying to understand the socio-economic status of the patient 3. Informing the cost of treatment 4. Providing financial assistance if needed	1. Trying to understand the socio-economic status of the patient 2. Informing the cost of treatment 3. Providing financial assistance if needed

A team of masters-level students at the Communication Department of a Bangladeshi university served as data collectors. When contacting each of the participants randomly chosen from the A2i database, they provided a brief explanation of the study objectives and procedures, as well as voluntary nature of their participation, and ensured them of the anonymity and confidentiality of the information gathered through the survey. After obtaining verbal informed consent from the eligible participants, the data collectors conducted the interview over the phone, which took 20–30 minutes to complete. The participants answered the 20 responsiveness questions using a 10-point scale, where 1 indicates negativity and 10 indicates positivity (Please see Supplementary File B for the tool). Thus, a higher score corresponds to greater ROP.

### Data management and analysis

All data analyses including data management, cleaning, missing value imputation, descriptive statistics, factor analysis and internal consistency checking of the factors were performed using Stata 16. We performed an exploratory factor analysis (EFA) to measure the factor structure and psychometric properties of the initial COVID-19 ROP-Scale comprising of 20 items. As only 53 and 36 participants answered the question related to examining patients (item 6) and offering financial assistance if needed (item 9), respectively, these two items were excluded from further analysis. The remaining 18 items were subjected to correlation matrix analysis, which revealed that item 7 (Trying to understand the socio-economic status of the patient) and item 8 (Giving idea about treatment cost), as well as item 10 (Explaining the cause of the disease to the patient) and item 11 (Explaining the disease diagnosis to the patient) were highly correlated (correlation coefficient value > 0.95) to each other. Therefore, to avoid the multicollinearity issue, items 7 and 10 were excluded, and EFA was performed again on the remaining 16 items. Since some of the values were missing, the maximum likelihood approach with expectation-maximisation (EM) algorithm was used to estimate the covariance matrix [10]. The *factormat* command in Stata was used to obtain the factor solution. Direct oblimin method was used for factor rotation [11] with the hypothesis that the factors are correlated, and Kaiser’s criteria (eigenvalue > 1 rule) were adopted to determine the number of factors to be retained in the model. A correlation cutoff value of 0.32 among factors was used to warrant oblique rotation as opposed to orthogonal rotation [12]. As only items with factor loadings ≥0.4 were retained [13], items 3 (Friendliness of the physician), 4 (Showing respect explicitly), 5 (Listening to patient’s complaints attentively), 8 (Giving idea about treatment cost), 11 (Explaining the diagnosis of disease to the patient), 12 (Explaining the prognosis of the disease to the patient), 13 (Explaining the treatment to the patient), 17 (Giving courage and reassurance) and 20 (Sense of humor) were excluded. Before EFA, we checked the suitability of data for factor analysis by conducting Bartlett’s test and Kaiser-Meyer-Olkin (KMO) test [14].

Confirmatory factor analysis (CFA) was performed to test the validity of the factors derived through the EFA analysis. Several criteria such as Chi-square test of overall model fit, Root mean square error of approximation (RMSE), Comparative fit index (CFI) and Tucker-Lewis index (TLI) were used to assess goodness of fit of the CFA model. A non-significant chi-square value where the null hypothesis is that the model fits well, RMSEA ≤0.08, CFI ≥ 0.90 and TLI ≥ 0.90 were considered as indication of acceptable model fit [15, 16]. Standardized path coefficients were presented. All statistical tests were performed considering a 5% level of significance.

**Fig. 1:**
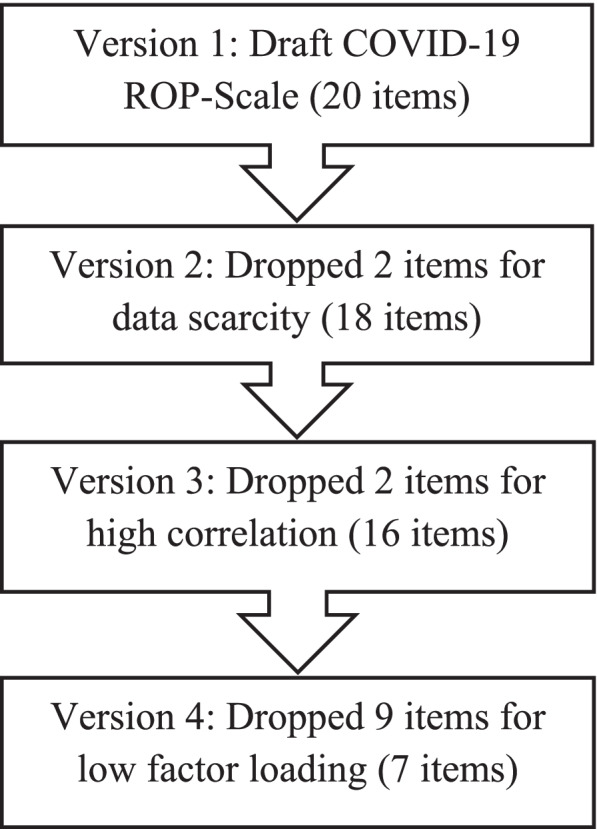
COVID-19 ROP-Scale validation process

Cronbach’s alpha was used to assess the internal consistency reliability of the three factors or domains. The corrected item-total correlation was also calculated. Concurrent validity of the 7-items COVID-19 ROP-Scale was assessed by examining Pearson’s correlation between the COVID-19 ROP-Scale score (i.e., the summed score of all items) and overall patient satisfaction, under the assumption that responsiveness would be positively correlated with satisfaction [17, 18]. Figure 1 shows the COVID-19 ROP-Scale validation process.

## Results

### Characteristics of sample

As can be seen from Table 2, the mean age and family size of the 213 participants (65% of whom were male) was 40 years and five members, respectively, and monthly income was 21,000 Bangladeshi Taka (USD 244). The participating patients were hospitalised for 9 days on average, and spent about 23,000 Bangladeshi Taka (USD 269) on their care. Majority of the participants (94%) received healthcare from a public hospital.

**Table 2:** Socio-demographic and health service characteristics of the participants

Background characteristics		
	**Mean**	**Standard Deviation**
Age (years)	40.43	14.47
Family members (number)	5.03	1.92
Monthly income (Bangladeshi Taka)	20,828.64 (USD ~ 244)	21,684.87 (USD ~ 254)
Number of days in hospital	9.24	4.73
Treatment expenditure (Bangladeshi Taka)	22,941.78 (USD ~ 269)	46,693.05 (USD ~ 548)
	**Frequency**	**Percentage**
Gender		
Male	139	65.26
Female	74	34.74
Education		
No education	6	2.82
Primary complete	13	6.10
Secondary complete (SSC and HSC)	98	46.01
Graduate and above	96	45.07
Religion		
Islam	172	80.75
Hinduism	40	18.78
Christianity	1	0.47
Marital Status		
Currently Married	181	84.98
Separated/ Deserted/ Divorced	6	2.82
Never married	26	12.21
Residence		
Urban	148	69.48
Rural	65	30.52
Occupation		
Farmer/agricultural worker	5	2.35
Business/ informal worker	20	9.39
Service holder/ government/ private formal job	139	65.26
Housewife	19	8.92
Day labourer	1	0.47
Student	8	3.76
Retired/ senior citizen	15	7.04
Unemployed	5	2.35
Others	1	0.47
Housing type (Number of rooms)		
Below 3	110	51.64
3–4	59	27.70
5 or More	44	20.66
Type of healthcare facility		
Public sector	201	94.37
Private sector	12	5.63
Severity of illness		
Mild	119	55.87
Moderate	72	33.80
Severe	22	10.33

### Content validity

The expert panel assessed the relevance and phrasing of the 20-item draft COVID-19 ROP-Scale and discussed any changes until reaching a consensus.

### Construct validity

#### Determining the number of factors to retain

The calculated KMO and Bartlett’s test was 0.84 with a *p* < 0.001. As per the Kaiser’s criteria (eigenvalue > 1 rule) three factors were considered to be relevant. The direct oblimin rotation procedure revealed that the factors are correlated (Supplementary File A).

#### Factor extraction and rotation

In the direct oblimin rotation analysis, the items ‘Explaining the preventive aspects of COVID-19 to the patient,’ ‘Encouraging patients to ask questions,’ and ‘Facilitating follow up’ were loaded heavily (with 0.48, 0.60, and 0.66 loadings, respectively) on Factor 1 (Informativeness). Similarly, items ‘Service-oriented, not business-like attitude’ and ‘Not being involved in illegal activities’ were loaded (with 0.70 and 0.97 loadings, respectively) on Factor 2 (Trustworthiness). Finally, items ‘Greetings by physician’ and ‘Engaging in social talk’ were loaded (with 0.54 and 0.87 loadings, respectively) on Factor 3 (Courteousness) (Table 3).

**Table 3:** Rotated pattern matrix of exploratory factor analysis

Items	Factor 1: Informativeness	Factor 2: Trustworthiness	Factor 3: Courteousness
Explaining the preventive aspects of COVID-19 to the patient	0.48		
Encouraging patients to ask questions	0.60		
Facilitating follow up	0.66		
Service-oriented, not business-like attitude		0.70	
Not being involved in illegal activities		0.97	
Greetings by physician			0.54
Engaging in social talks			0.87

**Fig. 2:**
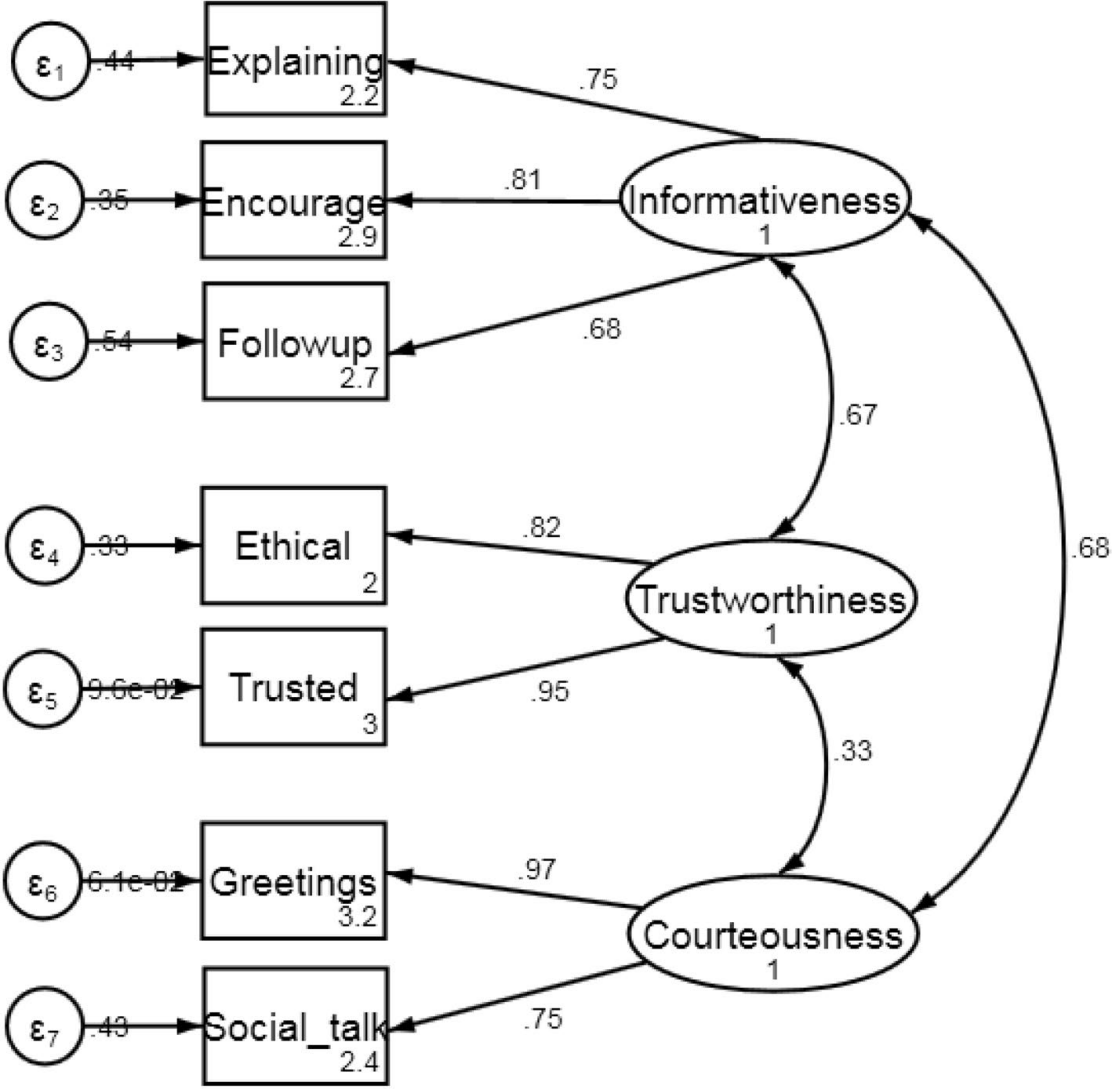
Final three-factor model for responsiveness of physicians during the COVID-19 pandemic. Note: All the standardized path coefficients (factor loadings) were significant (*p* < 0.001). Explaining: Explaining the preventive aspects of COVID-19; Encourage: Encouraging patients to ask questions; Followup: Facilitating follow up; Ethical: Service oriented not business-like attitude; Trusted: Not being involved in illegal activities; Greetings: Greetings by physician; Social_talk: Engaging in social talks

The CFA results with three-factors specified by the EFA involving 7 items indicated a better model fit with a Chi-square value, χ2 (11) = 12.80, *p*-value = 0.3064, RMSEA estimate of 0.028, CFI of 0.997 and TLI of 0.994 (Table 4). Figure 2 indicates the standardized path coefficients (factor loadings) and correlation coefficient values of the final three-factor CFA model that measures the underlying construct “responsiveness of physicians during the COVID-19 pandemic” in Bangladesh. All the factor loadings were significant (*p* < 0.001) and in expected direction (Fig. 2).

**Table 4:** Fit statistics from the three-factor confirmatory factor analysis

Model	χ^**2**^	df	***P***	RMSEA	CFI	TLI
Three-factor model: Informativeness, Trustworthiness and Courteousness	12.803	11	0.306	0.028	0.997	0.994

### Internal consistency

The internal consistency reliability of the three factors (Factor 1, Factor 2 and Factor 3) based on the Cronbach’s alpha coefficient values were 0.78, 0.59 and 0.84, respectively.

The final COVID-19 ROP-Scale included three factors, i.e., Informativeness, Trustworthiness and Courteousness (as shown in Table 5). The corrected item-total correlation ranged between 0.45 and 0.71. The Pearson’s correlation coefficient for the level of overall satisfaction and the responsiveness of the COVID-19 ROP-Scale score was 0.84, which was statistically significant at 5% level (2-tailed).

**Table 5:** The COVID-19 Responsiveness of Physicians Scale (ROP-Scale) with internal consistency measures and mean item score

Items (names slightly modified from the original)	Corrected item-total correlation	Cronbach’s alpha coefficient for domains	Mean item score	Mean domains score
**Factor 1: Informativeness**				
1. Explaining the preventive aspects of COVID-19 to the patient	0.67	0.78	6.92	7.28
2. Encouraging patients to ask questions	0.71		7.41	
3. Facilitating follow up	0.59		7.43	
**Factor 2: Trustworthiness**				
4. Service-oriented, not business-like attitude	0.45	0.59	6.63	7.85
5. Not being involved in illegal activities	0.67		7.97	
**Factor 3: Courteousness**				
6. Greetings by physician	0.65	0.84	7.75	7.44
7. Engaging in social talks	0.54		7.14	

### Responsiveness of physicians to hospitalised COVID-19 patients in Bangladesh

**Fig. 3:**
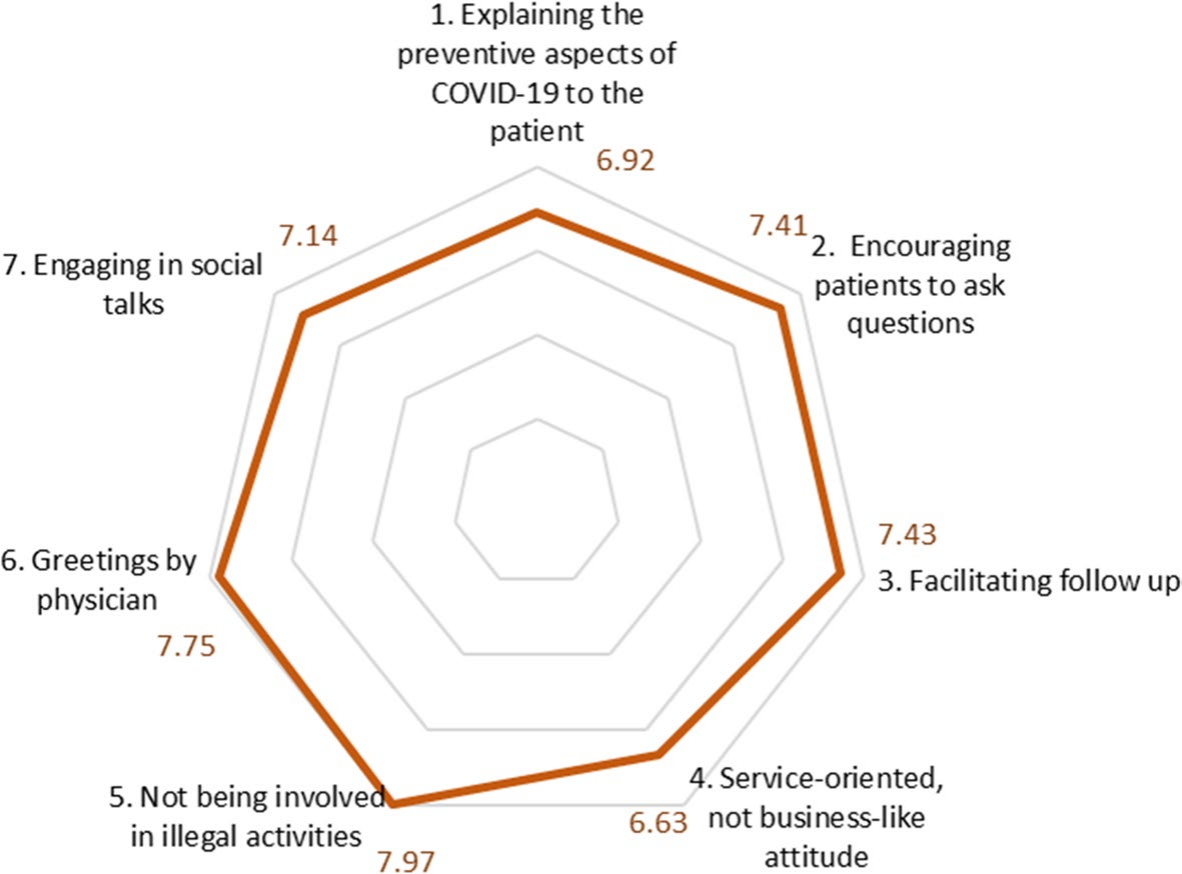
COVID-19 ROP-Scale scores

As can be seen from Table 5, the highest- and the lowest-scoring responsiveness domain are ‘Trustworthiness’ (mean domain score 7.85), and ‘Informativeness’ (7.28). The highest-scoring item was ‘Not being involved in illegal activities’ (7.97) and the lowest scoring ‘Service-oriented, not business-like attitude’ (6.63) (Fig. 3).

## Discussion

### Statement of principal findings

As a part of our study, we validated the 7-item COVID-19 ROP-Scale with three domains, namely, Informativeness, Trustworthiness, and Courteousness. The scale was found to be feasible, valid, and internally consistent. The concurrent validity analyses further confirmed that higher responsiveness of physicians was associated with greater patient satisfaction.

### Interpretation within the context of the wider literature

The main benefit of this scale stems from its feasibility, as it comprises of only 7 items, and it can be applied through exit interviews with the recent COVID-19 or similar infectious disease patients. While EFA is useful in psychometric studies for examining the dimensionality of the domains [19], the sample must be suitable for EFA, which according to Kaiser and Rice requires a statistically significant Bartlett’s test and a > 0.80 KMO statistic. They also provided an evaluation criterion, according to which our sample was ‘meritorious’ to perform a satisfactory factor analysis [14]. Internal consistency reliability of the COVID-19 ROP-Scale was ascertained through a reasonably high Cronbach’s alpha coefficient and corrected item-total correlation, based on the 0.70 and 0.35 cut-off values, as recommended by Taber [20] and Netemeyer [21], respectively.

Criterion validity is the extent to which the scale score is associated with a relevant criterion variable external to the scale. One of the types of criterion validity is the concurrent validity, which is measured by assessing the correlation between the score of the scale under development and the concurrently collected criterion variable [21]. In the present study, a correlation coefficient of ≥0.50 was considered acceptable and was well surpassed by the COVID-19 ROP-Scale. The high Pearson’s correlation coefficient (0.84) not only indicates a high concurrent validity, but also denotes the importance of responsiveness of physicians in achieving patient satisfaction, as shown by other authors [1, 17, 18, 22–25].

Empirical evidence further indicates that physicians in Bangladeshi inpatient settings scored high in ‘Trustworthiness’ but not so much in ‘Informativeness.’ This finding is aligned with the past application of ROP-Scale among the rural Bangladeshi outpatient settings, where ‘Gaining trust’ was the highest- and ‘Friendliness’ was the lowest-scoring domain [7]. Similar results were obtained in Lithuania, where the lack of information provided by health service providers was reported by patients as one of the main reasons for their dissatisfaction with the healthcare provision [26].

### Strengths and limitations

The main strength of this study is its conciseness and ease of completion, as all responses are required on a 10-point scale (rather than the 4-point Likert type scale used in the original ROP-Scale), which some patients may find more intuitive. This response format was also preferable as participants could select a number without having to read the corresponding description, and it avoids potential skewedness caused by the range of response options chosen (e.g. 5- versus 6-point Likert scale) [27, 28].

The main limitation of this study is that the test-retest and inter-rater reliabilities of this scale were not evaluated. This decision was deliberate, as the study participants had recently recovered from COVID-19 at the time of the survey, and we did not deem it appropriate to engage them in yet another round of questioning. Secondly, the COVID-19 pandemic and the consequent lockdowns in Bangladesh also imposed time and resource constraints on the research team. Finally, the overall Cronbach’s alpha was not calculated because it is not known whether the extracted three factors (Informativeness, Trustworthiness and Courteousness) could be loaded to a single general factor (Responsiveness of Physicians) which needs to be confirmed by higher-order CFA or Bi-factor model.

### Implications for policy, practice, and research

As the health system stewards attempt to improve the COVID-19 service delivery, they can use the present study findings to identify the gaps in service provision and develop a strategy for improving the responsiveness of physicians. This will not only enhance the service delivery but also increase patient satisfaction.

The data generated from this study and through future applications of the COVID-19 ROP-Scale can help determine and compare physician responsiveness in different geographic areas or healthcare settings, and among different patient populations, with the goal of addressing any identified gaps.

## Conclusion

As demonstrated in this study, the revised ROP-Scale is valid and can be used in the context of COVID-19 pandemic or similar infectious disease outbreaks in Bangladesh or similar low-resource settings. This is particularly useful at a time when many countries have started experiencing new waves of the pandemic. The COVID-19 ROP-Scale can help to amend past mistakes in health service provision, and improve care for the hospitalised COVID-19 patients or other patients suffering from similar conditions. This study can contribute to the national decision-making regarding hospital care, open up further avenues in the health policy and system research, and eventually improve the quality of care provided to all patients seeking hospital services in Bangladesh. Incorporating the learnings from this study, the medical education and in-service training for the doctors can be improved towards a more satisfied patient population.

## Supplementary Information

**Additional file 1. **Correlation matrix of the oblimin rotated common factors.

**Additional file 2. **Phone Exit Survey Tool.

## Data Availability

The datasets used and/or analysed during the current study are available from the corresponding author on reasonable request.

## Abbreviations

A2i: Aspire to Innovate
CFA: Confirmatory Factor Analysis
COVID-19: Coronavirus Disease 2019
EFA: Exploratory Factor Analysis
EM: Expectation-maximisation
GoB: Government of Bangladesh
ICT: Information and Communication Technology
KMO: Kaiser-Meyer-Olkin
ROP: Responsiveness of Physicians
