# Supplementary material for: Validation of Responsiveness of Physicians Scale (ROP-Scale) for hospitalised COVID-19 patients in Bangladesh
Source: BMC Health Serv Res. 2022 Aug 15;22:1040. doi: 10.1186/s12913-022-08413-4 (PMC9376893; doi:10.1186/s12913-022-08413-4)
Supplement: Supplementary file 1 — Additional file 1. Correlation matrix of the oblimin rotated common factors. [file 12913_2022_8413_MOESM1_ESM.docx]

**Correlation matrix of the oblimin rotated common factors**

| **Factors** | **Factor 1** | **Factor 2** | **Factor 3** | **Factor 4** | **Factor 5** | **Factor 6** | **Factor 7** | **Factor 8** | **Factor 9** | **Factor 10** |
| --- | --- | --- | --- | --- | --- | --- | --- | --- | --- | --- |
| **Factor 1** | 1 |  |  |  |  |  |  |  |  |  |
| **Factor 2** | 0.5455 | 1 |  |  |  |  |  |  |  |  |
| **Factor 3** | 0.4721 | 0.2639 | 1 |  |  |  |  |  |  |  |
| **Factor 4** | 0.4387 | 0.5118 | 0.4215 | 1 |  |  |  |  |  |  |
| **Factor 5** | 0.5154 | 0.3041 | 0.5764 | 0.5368 | 1 |  |  |  |  |  |
| **Factor 6** | 0.52 | 0.4098 | 0.3087 | 0.1794 | 0.2109 | 1 |  |  |  |  |
| **Factor 7** | 0.2439 | 0.4769 | 0.1243 | 0.3521 | 0.1443 | 0.2875 | 1 |  |  |  |
| **Factor 8** | 0.2007 | 0.2992 | -0.07854 | 0.2504 | -0.00856 | 0.1909 | 0.5829 | 1 |  |  |
| **Factor 9** | 0.3583 | 0.0547 | 0.3034 | 0.1026 | 0.1176 | 0.2707 | 0.0114 | -0.1357 | 1 |  |
| **Factor 10** | 0.1438 | 0.2369 | 0.1499 | 0.2007 | 0.0816 | 0.2504 | 0.3369 | 0.2296 | 0.1498 | 1 |
