# Supplementary material for: Validation of Responsiveness of Physicians Scale (ROP-Scale) for hospitalised COVID-19 patients in Bangladesh
Source: BMC Health Serv Res. 2022 Aug 15;22:1040. doi: 10.1186/s12913-022-08413-4 (PMC9376893; doi:10.1186/s12913-022-08413-4)
Supplement: Supplementary file 2 — Additional file 2. Phone Exit Survey Tool. [file 12913_2022_8413_MOESM2_ESM.docx]

Validation of Responsiveness of Physicians Scale (ROP-Scale) for Hospitalized Covid-19 Patients in Bangladesh

Phone Exit Survey Tool

**Date of data collection:** ___/___/____ **Questionnaire ID:** |___|___|___|___|

**Section A: Socio Demographic Characteristics**

1. **Name of respondent (verify from database):** ____________________________________________________________
2. **Contact information/ mobile number (from database):** ______________________________________
3. **Address (verify from database):**

a. Upazila:

b. District:

c. Division:

1. **Age (verify from database):**

_______ years

1. **Sex (from database):**

a. Male

b. Female

1. **Education:**

a. No education

b. Primary (1-5)

c. Secondary (6-10)/ Higher Secondary (11-12)

d. Higher (Batchelor, Masters)

1. **Occupation (from database):**

a. Agricultural

b. Business/ informal worker

c. Service holder/ government/ private formal job

d. Housewife

e. Day labor

f. Driver (Rickshaw/ auto rickshaw/ Van/ CNG/ Bus/ Truck/ Car)

g. Student

h. Retired/ aged people

i. Unemployed

j. Others __________________________________

1. **Religion:**

a. Islam

b. Hinduism

c. Buddhism

d. Christianity

e. Others __________________________________

1. **Marital status:**

a. Currently Married

b. Separated/ Deserted/ Divorced

c. Never married

1. **Number of family member/ Family size:**

_______ persons

1. **Current living residence:**

a. Urban

b. Rural

1. **Number of rooms in current residence/ home:**

_______ rooms

1. **Monthly income of respondent:**

_______ Taka

1. **Test date (from database):**

Day…..Month…..Year……..

1. **Admission date (from database):**

Day…..Month…..Year……..

1. **Discharge date:**

Day…..Month…..Year……..

1. **Number of days stayed in hospital (verify from database):**

_______ days

1. **Institute ID (from database):**

_______

1. **Type of healthcare facility:**

a. Public sector

b. Private sector

1. **Severity (from database):**

a. Mild

b. Moderate

c. Severe

1. **How much did you have to spend for your treatment?**

_______ Taka

1. **Level of overall satisfaction from the services received from the doctors in the hospital (expressed in 1 to 10 where 1 means low and 10 means high satisfaction): [This question should be asked at the end of the survey]**

1---2---3---4---5---6---7---8---9---10

**Section B: Responsiveness of Physicians Scale (ROP-Scale) Items**

## Begnning part

1. A responsive doctor should greet and welcome the patient or make the patient comfortable so that the patient can easily express their problems. Did the doctor greet and welcome the patient, or made them comfortable so that they could express their problems easily? For example, giving salam to the patient (Saying Adab/Nomoskar to the Hindus and others as per their religion), asking the name of the patient and calling in his name, asking his well beings (how are you/what’s the matter), appropriate salutation like mother, father, brother, sister, sister-in-law, saluting ‘Babu’ (in case of children), Sweety etc. Asking to take a seat, asking whether he had his breakfast, asking about his residence and profession, smiling at him; to shake hands with him; showing respect to the aged person by standing up etc. and accept the patient cordially, reply the patient’s greetings and ask the patient’s well being. How much would you give between 1 and 10 to the doctor in terms of greeting and welcomeing the patient?

1---2---3---4---5---6---7---8---9---10---Not Applicable

2. A responsive doctor should not only listen to their problem but also do some social talks and listen they do any social talk. During your contact with the doctor did the doctor engage in social talk or only clinical talks? Following social talks might be done, the patient’s profession, education, children, family members, weather etc. How much would you give between 1 and 10 to the doctor in terms of conducting social talks?

1---2---3---4---5---6---7---8---9---10---Not Applicable

3. A responsive doctor should express some gestures of friendliness or speak in a friendly ton. Did the doctor show a friendly gesture or speak in a friendly tone? Some friendly gestures may include, remembering the name and face of the patient and calling him by name (here ‘calling by name’ means calling by name of the patient in a friendly manner); asking or making comment about an event of the patient’s family; praising the patient (about clothing or anything else); asking for an opinion of the patient about anything (weather, politics etc.). How much would you give between 1 and 10 to the doctor in terms of expressing friendy gestures or speaking in a friendly tone?

1---2---3---4---5---6---7---8---9---10---Not Applicable

4. A responsive doctor should show respect in all their conducts during the consultation to the patient. Did the doctor show respect in all conducts during the consultation with you? The example of showing respect to the patient may include giving honor to an aged patient by standing up, helping an aged patient to sit down, giving Salam or at least replying when patients give Salam, talking softly with the patient etc. On the other hand, examples showing disrespect may include, bargaining for money, using bad words, denying to provide treatment etc.; stopping the patient in the middle; talking in an authoritative tone, misbehaving, scolding etc.; getting the patient out of the room; “Do you know more than me? Then why did not you become a doctor?”-Telling such etc. How much would you give between 1 and 10 to the doctor in terms of showing respect in all conducts during consultation?

1---2---3---4---5---6---7---8---9---10---Not Applicable

## History taking

5. A responsive doctor should listen to patients attentively with patience and that would be expressed by doctor’s behavior. Did the doctor listen to you attentively with patients and expressed the attention in gesturs? Behaviors indicating attentiveness and patience may include, shaking head while talking, looking at the patient, asking questions to learn more, variation in tone, smiling face, some interest expressing words (e.g., Ok, hm etc.) etc. How much would you give between 1 and 10 to the doctor in terms of listening attentively with patience?

1---2---3---4---5---6---7---8---9---10---Not Applicable

## Examining

6. A responsive doctor should do the necessary physical examination with care. Did the doctor conduct physical examinations, if any, with care? The example of examining the patient with care may may include, telling the patient politely to fold up their sleeves, telling the patient that what the doctor is going to do, conduct the whole procedure politely, etc. How much would you give between 1 and 10 to the doctor in terms of conducting physical examinations with care? **[Use ‘Not applicable’ if the doctor did not conduct any physical examination]**

1---2---3---4---5---6---7---8---9---10---Not Applicable

## Prescribing

7. A responsive doctor should try to understand the socio-economic condition of the patient before providing treatment. Did the doctor try to understand your socio-economic condition? Example of trying to understand the financial condition of the patient may be: Asking the patient directly about their income or whether they would be able to bear the treatment cost; Asking them indirectly (such as asking their profession). Beside these, it might be guessed by observing the patient’s conversation, behavior and clothing. How much would you give between 1 and 10 to the doctor in terms of trying to understand the socio-economic condition of the patient? **[Use ‘Not applicable’ if, for any reason, it is not possible to rate the doctor in this regard or it was not possible to understand]**

1---2---3---4---5---6---7---8---9---10---Not Applicable

8. A responsive doctor should give patients a clear idea about treatment cost before starting treatment. Did your doctor give a clear idea about the treatment cost? Example of giving idea about treatment cost may include, how much would be needed to complete the treatment; how long the treatment may continue; what impact the patient would be able to put on his ability of income during and after receiving treatment, etc. How much would you give between 1 and 10 to the doctor in terms of giving a clear idea about treatment cost? **[Use ‘Not applicable’ if, for any reason, it is not possible to rate the doctor in this regard]**

1---2---3---4---5---6---7---8---9---10---Not Applicable

9. A responsive doctor should help patients if they become unable to bear the cost of the treatment. If you were unable to bear the treatment cost, did the doctor help you in any manner? For example, prescribing low cost antibiotics, taking less or no consultation fee (in case of private doctors), providing financial assistance to the poor patients, helping in getting free medicines from the hospital (in case of government doctors), giving time and advice to obtain money for treatment, trying to focus on the history and physical examination to avoid investigation, prescribing the essential tests only, deducting the commission paid to the doctor for each test, recommending the treatment method that saves money (to meet the nutritional needs from domestic sources, etc.) and so on. How much would you give between 1 and 10 to the doctor in terms of helping the patient if they are unable to bear the treatment cost? **[If the patient is able to bear treatment cost or does not require any help or for any reason it is considered inapplicable, please fill in the ‘Not applicable' field]**

1---2---3---4---5---6---7---8---9---10---Not Applicable

## Explaining

10. A responsive doctor should explain the **cause** of the disease like why the disease occurred, what may be the causes of the disease, etc., they would do it on their own (that means they would not give responsibility to assistant, pharmacist, rather would tell it themselves) and would ask whether the patient has understood. Did you doctor explain the cause of the disease him/herself and ask whether you understood the cause of the disease? How much would you give between 1 and 10 to the doctor in terms of explaining the cause of the disease? **[If in the current context there is no need or situation to explain the cause of the disease (such as, the cause is reasonably believed to be already known) fill in the ‘Not Applicable’ field]**

1---2---3---4---5---6---7---8---9---10---Not Applicable

11. A responsive doctor should explain in details about the **diagnosis** (that means the name of the disease) of their diseases (However, it should be told in such a way that it does not create panic), they would do it on their own (that means they would not give responsibility to assistant, pharmacist, rather would tell it themselves) and would ask whether the patient has understood. Did you doctor explain the diagnosis of the disease him/herself and ask whether you understood the diagnosis of the disease? How much would you give between 1 and 10 to the doctor in terms of explaining the diagnosis of the disease? **[If in the current context there is no need or situation to explain the diagnosis of the disease (such as, the diagnosis is reasonably believed to be already known) fill in the ‘Not Applicable’ field]**

1---2---3---4---5---6---7---8---9---10---Not Applicable

12. A responsive doctor should explain in details about the **severity of the disease, prognosis** (recovery, consequence etc.) etc., they would do it on their own (that means they would not give responsibility to assistant, pharmacist, rather would tell it themselves) and would ask whether the patient has understood. Did you doctor explain the severity and prognosis of the disease him/herself and ask whether you understood the severity and prognosis of the disease? How much would you give between 1 and 10 to the doctor in terms of explaining the severity and prognosis of the disease?

1---2---3---4---5---6---7---8---9---10---Not Applicable

13. A responsive doctor should explain in details about the **treatment** of their diseases like which medicines have been given and why, how to take those medicines etc., they would do it on their own (that means they would not give responsibility to assistant, pharmacist, rather would tell it themselves) and would ask whether the patient has understood. Did you doctor explain the treatment of the disease him/herself and ask whether you understood the treatment of the disease? How much would you give between 1 and 10 to the doctor in terms of explaining the treatment of the disease?

1---2---3---4---5---6---7---8---9---10---Not Applicable

14. A responsive doctor, along with the treatment of the disease, should also explain in details about **diet**, which foods are allowed and which are forbidden, prevention of the disease for which s/he has gone to the doctor, how to remain away from it etc. as well as lifestyle modification, preventive advice etc., they would do it on their own (that means they would not give responsibility to assistant, pharmacist, rather would tell it themselves) and would ask whether the patient has understood. Did you doctor explain the diet of the disease him/herself and ask whether you understood the diet of the disease? How much would you give between 1 and 10 to the doctor in terms of explaining the diet of the disease?

1---2---3---4---5---6---7---8---9---10---Not Applicable

15. Some of the behavior of the doctor discourages patients to ask questions. A responsive doctor should not do anything that may discourage the patient to ask question; rather the doctor should behave such that would provide courage and encourage patients to ask questions. Did your doctor refrain from anything that may discourage you to ask question and do things that may encourage you to ask question? Examples of discouraging behavior may include, repeatedly looking at the clock, giving reminder to the patient to be short, writing prescription while answering the question, ultra-seriousness, answering very shortly (in one word), answering in nagging tone, answering in a neglectful manner, etc. On the contrary, examples of encouraging conduct may include, answering questions with a smiling face, carefully listening to the questions, etc. How much would you give between 1 and 10 to the doctor in terms of encouraging the patient to ask question?

1---2---3---4---5---6---7---8---9---10---Not Applicable

## Leaving

16. A responsive doctor should facilitate post treatment follow-up and give them a clear follow-up plan. Did the doctor give you a clear follow-up plan? For example, when the patient would meet the doctor again; in which case the patient should contact the doctor before; if necessary, how the patient can reach the doctor; providing mobile number to the patient; telling about follow-up costs; to write down what the patient should bring at the time of follow-up (or at least tell); telling to inform the doctor immediately if any of the side effects of treatment arise etc. How much would you give between 1 and 10 to the doctor in terms of facilitating follow-up?

1---2---3---4---5---6---7---8---9---10---Not Applicable

## Throughout consultation

17. A responsive doctor should give courage and reassurance to the patient through his/her words and behavior. Did your doctor give you courage and reassurance to assuage your worry, fear, stress of the disease? Reassurance expressing speech and behavior may include, saying things like, you have no problem, you will be all right, nothing has happened to you, there is nothing to be worried, I would be able to cure your disease, inshallah, etc.; doing things like, putting hands on the shoulder of the patient, giving them courage by holding their hand, giving courage by putting hand on the body; etc. How much would you give between 1 and 10 to the doctor in terms of giving courage and reassurance?

1---2---3---4---5---6---7---8---9---10---Not Applicable

18. A responsive doctor should be service-oriented instead of business-oriented. Did you find your doctor to be service-oriented or business oriented? Examples of being service-oriented may include, asking the patient’s ability to bear the cost of treatment, if necessary, assisting the patient in getting low-cost medical care and so on. Examples of being business-oriented may include, telling the patient to do test from any specific diagnostic center, encouraging to buy medicines of a specific pharmaceutical company, taking money from patients forcibly, encouraging the patient under consultation of a public sector doctor to go to a private clinic, etc. How much would you give between 1 and 10 to the doctor in terms of being service-oriented?

1---2---3---4---5---6---7---8---9---10---Not Applicable

19. A responsive doctor should refrain from any illegal or unethical activity. Did you find your doctor staying away from any illegal or unethical activity during your treatment? Examples of such illegal activities may be: Taking money from patients against free services; bringing patients with the help of brokers; presence of brokers around the chamber (Doctors did not take any step to stop this); collusion with diagnostic centers, accepting gift from medical representative and prescribing medicine of that company, taking advantage from brokers, etc. How much would you give between 1 and 10 to the doctor in terms of their refraining form illegal or unethical activities? **[Please fill out ‘Not applicable’ if the patient is reasonable unable to give opinion on this issue]**

1---2---3---4---5---6---7---8---9---10---Not Applicable

20. A responsive doctor should have sense of humor and provide treatment by becoming easy with patients using his sense of humor. Did your doctor, during your treatment, show sense of humor or at least stay with a smiling face? How much would you give between 1 and 10 to the doctor in terms of their sense of humor or staying with a smiling face?

1---2---3---4---5---6---7---8---9---10---Not Applicable

Last question, from Section A/ Section A

1. **Level of overall satisfaction from the services received from the doctors in the hospital (expressed in 1 to 10 where 1 means low and 10 means high satisfaction):**

1---2---3---4---5---6---7---8---9---10

END
